# Association Among Cystic Volume, Intracystic Pressure, and Histopathological Changes in the Liver in Patients With Choledochal Cyst

**DOI:** 10.7759/cureus.50208

**Published:** 2023-12-09

**Authors:** Fatima Tul Jannat, K. M. Didarul Islam, Mahmud Hasan Mostofa Kamal, Bishnu Pada Dey, Noor Mahammad, Umme Habiba Dilshad Munmun, Jannatul Nayeema Tonny, Md. Shahinur Rahman, Md. Ruhul Amin, A. K. M. Zahid Hossain

**Affiliations:** 1 Department of Pediatric Surgery, Bangabandhu Sheikh Mujib Medical University, Dhaka, BGD; 2 Department of Pediatric Surgery, Nobojatok-Shishu and General Hospital, Dhaka, BGD; 3 Department of Radiology & Imaging, Bangabandhu Sheikh Mujib Medical University, Dhaka, BGD; 4 Department of Pathology, Bangabandhu Sheikh Mujib Medical University, Dhaka, BGD; 5 Department of Pediatric Surgery, Armed Forces Medical Institute, Dhaka, BGD; 6 Department of Pediatric Surgery, Rangpur Medical College and Hospital, Rangpur, BGD

**Keywords:** metavir, liver fibrosis, intracystic pressure, cystic volume, choledochal cyst

## Abstract

Background

Choledochal cyst is a congenital cystic dilatation of the biliary tree. Various aspects of pathophysiology are unclear, particularly with reference to intracholedochal cystic pressure (ICCP) and liver histopathology. This study aimed to determine the relationship among cystic volume, ICCP, and histopathological changes in the liver in patients with choledochal cysts.

Methods

This cross-sectional study was carried out among 21 patients diagnosed with choledochal cysts, who attended the Department of Pediatric Surgery at Bangabandhu Sheikh Mujib Medical University (BSMMU) from April 2021 to August 2022. Cystic volume was measured pre-operatively using ultrasonography, while ICCP was measured per-operatively with a pressure gauge. Liver histology was assessed through an intraoperative liver biopsy and graded using the meta-analysis of histological data in viral hepatitis (METAVIR) scoring system. The data were analyzed using SPSS version 25.0 (IBM Corporation, Armonk, New York). Frequency and percentages were calculated to present categorical variables, and for quantitative variables, mean, standard deviation (SD), median, and interquartile range (IQR) were calculated. Fisher’s exact tests were performed to determine the association between cystic volume, ICCP, and the grading of hepatic fibrosis. A p-value of <0.05 was considered statistically significant.

Results

The age of the patients ranged from 1 to 12 years, with a mean of 5.0±3.4 years. The male-to-female ratio was 1:4.3. Type I cysts were the most prevalent (71.4%). The median and IQR for cystic volume were 3.4 ml and 1.1-8.2 ml, respectively. The median and IQR for ICCP were 7.46 mmHg and 4.67-9.33 mmHg, respectively. The most frequent grade of fibrosis was F1 (38.1%), followed by F2 (23.8%) and F3 (23.8%). A negative relationship between cystic volume and ICCP was observed, which was statistically significant (p=0.008). A positive relationship was also observed between ICCP and the grading of liver fibrosis, which was statistically significant (p=0.002). Although a negative correlation between cystic volume and grading of liver fibrosis was noted, it was not statistically significant (p=0.198).

Conclusions

This study reveals significant associations between cystic volume, ICCP, and the grading of liver fibrosis in patients with choledochal cysts. Smaller volume cysts may exhibit higher ICCP, resulting in more pronounced fibrotic changes in the liver.

## Introduction

Choledochal cysts are rare anomalies in the biliary tree characterized by the dilatation of the extrahepatic and/or intrahepatic bile ducts or both [1]. Symptoms associated with choledochal cysts may include jaundice, abdominal pain, a palpable mass, and frequently, occurrences of pancreatitis or cholangitis [2]. Without early diagnosis and proper management, choledochal cysts can lead to significant morbidity and mortality [1].

The incidence of choledochal cysts in the western population is approximately 1 in 100,000-150,000 live births [3]. In Asian populations, the incidence is notably higher, with a reported rate of 1 in 1,000 [4]. Typically, choledochal cysts are diagnosed during childhood, with around 25% identified in adulthood [5]. These cysts are more prevalent in females compared to males, with a female-to-male ratio ranging from 3:1 to 4:1 [6].

The prognosis following the excision of a choledochal cyst is usually excellent, yet it is contingent on various factors such as the patient's age, cyst type, histologic features, and site [7]. While most patients with choledochal cysts have a favorable prognosis with timely intervention, a subset may face complications, including liver fibrosis, leading to prolonged liver dysfunction even after undergoing definitive surgery [8]. Patients with a choledochal cyst often exhibit significant pathological changes in the liver [9,10].

Several aspects of the etiology and pathophysiology of choledochal cysts remain unclear, particularly concerning liver histology and intracholedochal cystic pressure (ICCP). The significance of liver histology has not been adequately emphasized in the treatment of choledochal cysts [5]. A hypothesis posits that a tense cyst with elevated pressure may induce backpressure changes, subsequently affecting liver pathology. Limited literature has attempted to address this issue. Therefore, this study is designed to determine the relationship between the cyst's volume, ICCP, and histopathological changes in the liver.

## Materials and methods

Study design and settings

This cross-sectional study was carried out at the Department of Pediatric Surgery, Bangabandhu Sheikh Mujib Medical University (BSMMU), Dhaka, spanning from April 2021 to August 2022. Pediatric patients diagnosed with choledochal cysts undergoing surgery were the study population. A total of 21 patients were selected consecutively for this study.

Eligibility criteria

Eligible participants were patients under 18 years of age diagnosed with type I and type IV choledochal cysts and willing to undergo surgery. Conversely, patients were excluded if they presented with a ruptured or decompressed choledochal cyst or if they refused to participate in the study.

Operational definitions

Cyst Volume

The cyst volume was assessed pre-operatively using ultrasonography. Cystic volume was categorized into two groups based on the median cystic volume. Volumes less than the median value were considered low-volume cysts, while volumes equal to or greater than the median value were classified as high-volume cysts.

Intracholedochal Cystic Pressure

ICCP was measured intraoperatively using a device comprising an interconnected cannula and pressure gauge. ICCP was categorized into two groups based on the median ICCP. ICCP values less than the median were considered low-pressure cysts, while values equal to or greater than the median were classified as high-pressure cysts.

Grade of Hepatocellular Damage

The meta-analysis of histological data in viral hepatitis (METAVIR) score system [11] was used for grading hepatocellular damage (Table 1). Patients were categorized based on the degree of liver fibrosis into two groups: the mild fibrosis group (F0-F1) and the moderate-to-severe fibrosis group (F2-F4).

**Table 1:** METAVIR score system and fibrosis stage. METAVIR: meta-analysis of histological data in viral hepatitis.

METAVIR score system	Fibrosis stage
F0	No fibrosis can be detected
F1	Fibrosis exists with the expansion of portal zones
F2	Fibrosis exists with the expansion of most portal zones and occasional bridging
F3	Fibrosis exists with the expansion of most portal zones, marked bridging, and occasional modules
F4	Presence of cirrhosis

Study procedure

**Figure 1:**
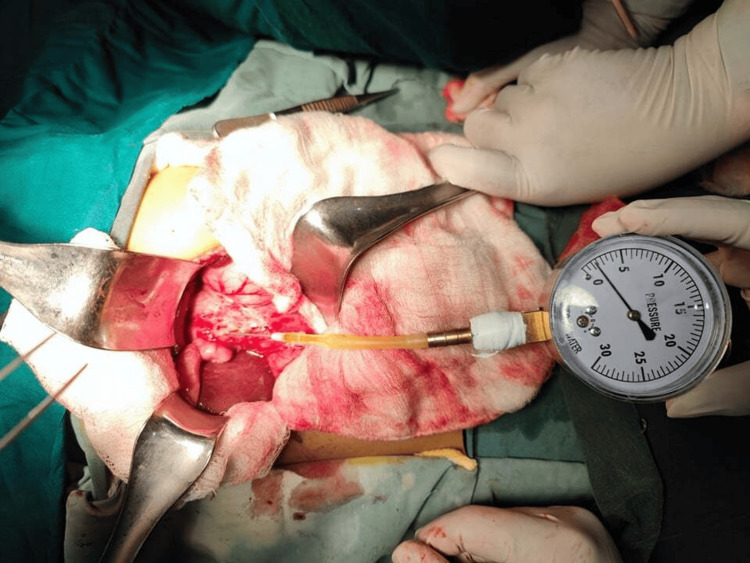
Measurement of intracystic pressure by a pressure gauge.

The clinical history, physical examination, and initial investigation reports (including magnetic resonance cholangiopancreatography (MRCP) reports) of the patients were documented. Following case selection, informed consent was obtained from the parents of the participating patients. Ultrasonography was conducted at the Department of Radiology and Imaging at BSMMU to assess the cystic volume. All children underwent surgical exploration. ICCP measurements were taken immediately after surgical access and before any mobilization of the liver and biliary tree. A pressure gauge connected with a needle was employed for ICCP measurement (Figure 1). Liver biopsy samples were collected and sent to the Department of Pathology at BSMMU.

Statistical analysis

Data analysis was performed using Statistical Package for Social Sciences (SPSS) version 25.0 (IBM Corporation, Armonk, New York). Descriptive statistics, including frequency and percentages, were calculated to present all categorical variables, such as sex and age groups, with age expressed as mean±SD. Cyst volume and ICCP were presented with median and interquartile range (IQR). Scatter plots were generated to illustrate the correlation between cystic volume, ICCP, and liver fibrosis. Fisher’s exact tests were conducted to determine the association between cystic volume, ICCP, and liver fibrosis. A p-value less than 0.05 was considered statistically significant.

Ethical considerations

Ethical clearance for the study was obtained from the Institutional Review Board (IRB) of BSMMU before the commencement of this study. After the committee approved the research protocol, permission for the study was obtained from the Department of Pediatric Surgery, BSMMU. Informed written consent was obtained from all the parents of the participants.

## Results

Demographic characteristics

The age ranged from 1 to 12 years, with a mean of 5.0±3.4 years. The majority of participants fell within the three to five years (38.1%) and more than five years (38.1%) age groups. The male-to-female ratio was 1:4.3 (Table 2).

**Table 2:** Distribution of the patients by age and sex. SD: standard deviation.

Demographic characteristics	Number	Percentage	
Age (years)			
Up to 2	5	23.8	
3-5	8	38.1	
More than 5	8	38.1	
Mean±SD	5.0±3.4		
Minimum-maximum	1-12		
Sex			
Male	4		19.0
Female	17		81.0
Total	21		100.0

Type of cyst

There were 15 cases of type I (71.4%) and six cases of type IV (28.6%).

Cystic volume and intracystic pressure

**Figure 2:**
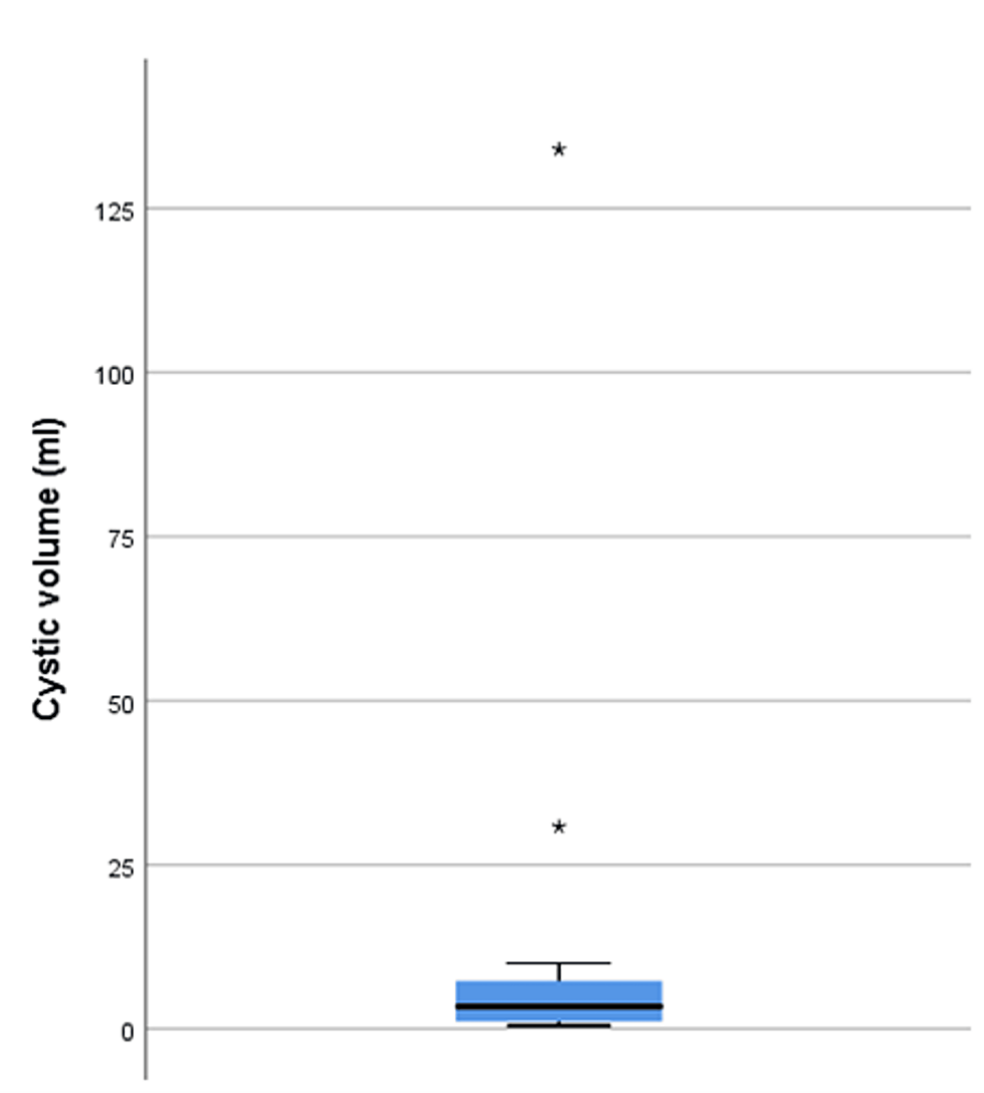
A box-and-whisker plot presenting the cystic volume of the patients. The figure shows the distribution of the sample by displaying the median and interquartile range of cystic volume, with asterisks signifying the outliers.

**Figure 3:**
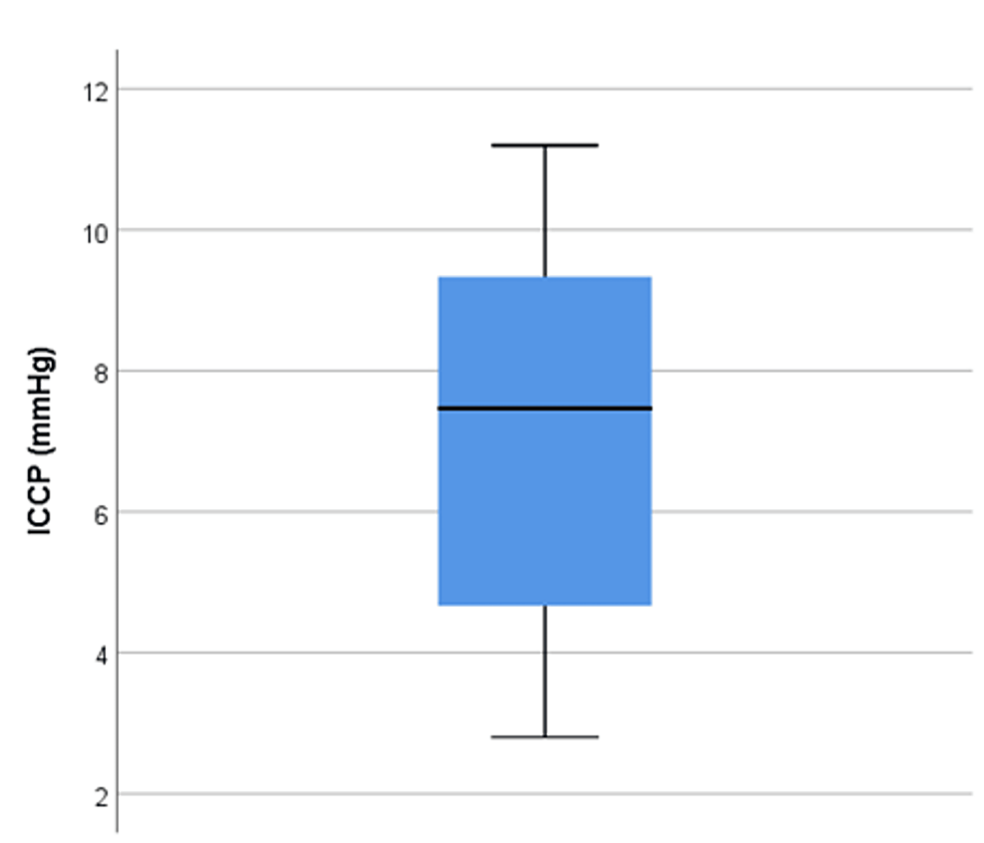
A box-and-whisker plot presenting the intracystic pressure of the patients. The figure shows the distribution of the sample by displaying the median and interquartile range of intracystic pressure. ICCP: intracystic pressure.

The volume ranged from 0.5 to 134 ml. The median and IQR were 3.4 ml and 1.1-8.2 ml, respectively (Figure 2). The ICCP ranged from 2.80 to 11.20 mmHg. The median pressure and IQR were 7.46 mmHg and 4.67-9.33 mmHg, respectively (Figure 3).

Grading of liver fibrosis

There were eight cases of F1 (38.1%), five cases of F2 (23.8%), and five cases of F3 (23.8%). Only three cases had no fibrosis (14.3%) (Table 3).

**Table 3:** Grading of liver fibrosis of the patients. METAVIR: meta-analysis of histological data in viral hepatitis.

METAVIR score	Number	Percentage
F0	3	14.3
F1	8	38.1
F2	5	23.8
F3	5	23.8
Total	21	100.0

Association between cystic volume and intracystic pressure

**Figure 4:**
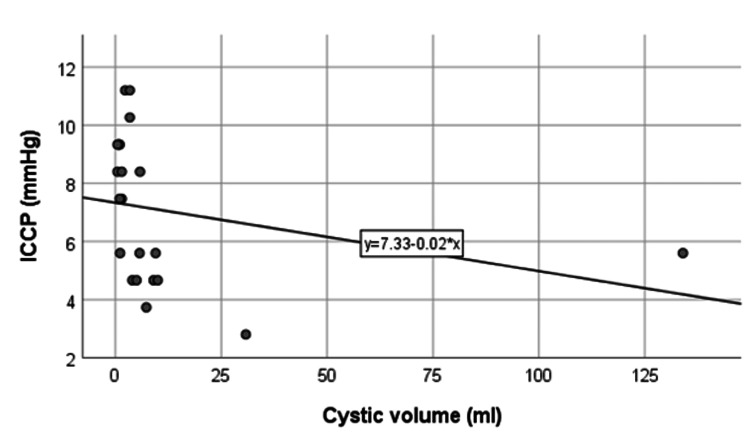
Correlation between cystic volume and intracystic pressure. ICCP: intracystic pressure.

Figure 4 illustrates the correlation between cystic volume and ICCP. There was a negative correlation between cystic volume and ICCP, i.e., ICCP decreased with the increase of cystic volume and vice versa. Table 4 demonstrates that the proportion of ICCP was higher when the cystic volume was <3.4 ml (88.9%) compared to cystic volume ≥3.4 ml (25.0%). This association was statistically significant (p=0.008).

**Table 4:** Association between cystic volume and intracystic pressure. p-value obtained from Fisher’s exact test.

Cystic volume	Intracystic pressure		p-value
	<7.46 mmHg	≥7.46 mmHg	
<3.4 ml	1 (11.1)	8 (88.9)	
≥3.4 ml	9 (75.0)	3 (25.0)	0.008
Total	10 (47.6)	11 (52.4)	

Association between intracystic pressure and grading of liver fibrosis

**Figure 5:**
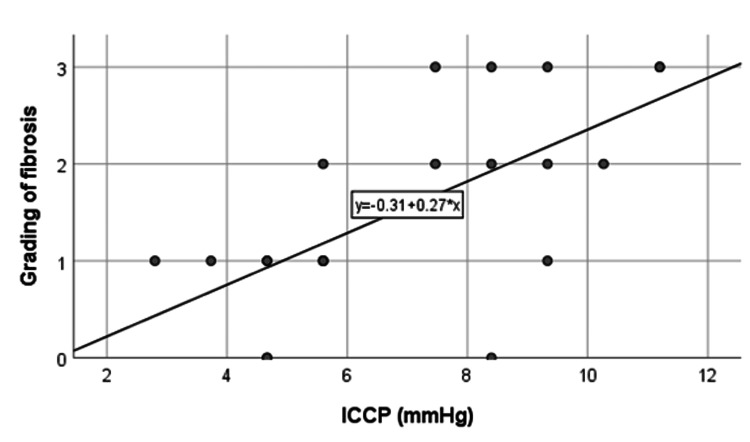
Correlation between intracystic pressure and grading of liver fibrosis. ICCP: intracystic pressure.

In Figure 5, a positive correlation is evident between ICCP and liver fibrosis grading, denoting an increase in fibrosis with elevated ICCP. Table 5 reveals a higher proportion of mild-grade fibrosis with lower ICCP (<7.46 mmHg) and a greater prevalence of moderate-severe fibrosis with higher ICCP (≥7.46 mmHg). This association is statistically significant (p=0.002), indicating the impact of ICCP on liver fibrosis progression.

**Table 5:** Association between intracystic pressure and grading of liver fibrosis. p-value obtained from Fisher’s Exact test.

Intracystic pressure	Grading of liver fibrosis		p-value
	F0-F1	F2-F3	
<7.46 mmHg	9 (90.0)	1 (10.0)	
≥7.46 mmHg	2 (18.2)	9 (81.8)	0.002
Total	11 (52.4)	10 (47.6)	

Association between cystic volume and grading of liver fibrosis

**Figure 6:**
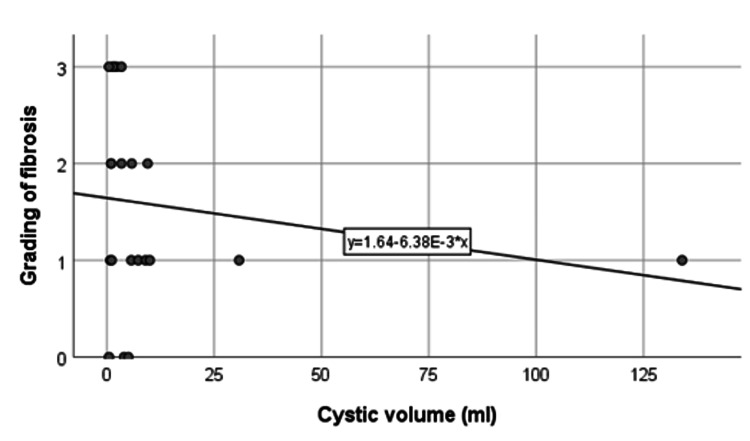
Correlation between cystic volume and grading of liver fibrosis.

In Figure 6, a negative correlation is evident between cystic volume and liver fibrosis grading, revealing higher fibrosis levels with decreased cystic volume. Table 6 shows a lower proportion of mild-grade fibrosis (F0-F1) with smaller cystic volumes (<3.4 ml) and a lower prevalence of moderate-severe fibrosis (F2-F3) with larger cystic volumes (≥3.4 ml). However, the association between cystic volume and grading of liver fibrosis was not statistically significant (p=0.198).

**Table 6:** Association between cystic volume and grading of liver fibrosis. p-value obtained from Fisher’s exact test.

Cystic volume	Grading of liver fibrosis		p-value
	F0-F1	F2-F3	
<3.4 ml	3 (33.3)	6 (66.7)	
≥3.4 ml	8 (66.7)	4 (33.3)	0.198
Total	11 (52.4)	10 (47.6)	

## Discussion

This study sought to explore the complex relationships among cystic volume, ICCP, and hepatic histopathology in choledochal cyst patients. The findings of the study contribute valuable insights to the understanding of this congenital anomaly.

The age of the participants in this study aligns with those of previous studies, wherein most patients presented at a young age [9,12-14]. Females comprised more than three-fourths of the study participants, with a male-to-female ratio of 1:4.3. Existing literature, particularly a recent review, consistently indicates a higher prevalence of biliary anomalies, e.g., atresia and choledochal cysts in females [15].

Type I cysts are the most common, constituting 80-90% of choledochal cysts [16]. The present study concentrated on type I and type IV cysts, enabling intraoperative ICCP measurements. Type I cysts predominated in this study, constituting 71.4% of cases. This distribution is in harmony with existing literature, which commonly reports type I cysts as the most prevalent among choledochal cyst variants [1,5].

The observed negative correlation between cystic volume and ICCP is a novel discovery, implying that smaller cysts may experience higher ICCP. Sharma et al. also presented a similar relationship between cystic volume and ICCP [12]. The relationship between cystic volume and ICCP can be explained by the principle that the pressure within a sphere is connected to both its volume and surface tension. Pressure and volume are inversely proportional; thus, an increase in volume leads to a decrease in pressure and vice versa. In a cyst connected to a system, back pressure effects are anticipated at both ends. In choledochal cysts, large cysts are expected to have low pressure and small-volume cysts are expected to have high pressure. Pressure effects on the ends are particularly expected, especially when the distal end is constrained by an extended common channel, thereby maximizing backpressure effects on the liver and cyst wall. This association may have implications for disease progression and warrants further exploration.

Liver fibrosis is prevalent in pediatric choledochal cyst patients, with an estimated incidence of 35% to 66.7% based on existing literature [17]. The present study echoes this trend, revealing varying degrees of fibrosis: 38.1% F1, 23.8% F2, 23.8% F3, and 14.3% without fibrosis. This aligns with Fumino et al.'s study findings [8], corroborating the consistency of liver fibrosis prevalence in this patient population.

The substantial positive correlation between ICCP and hepatic fibrosis grading is notable, suggesting the potential role of ICCP in influencing liver fibrotic changes. In a study, Sharma et al. found ICCP directly correlated with parenchymal changes such as hepatocellular damage [12]. The link between elevated ICCP and liver fibrosis implies that pressure measurement can act as a direct predictor of associated liver histopathological changes. This suggests that obstruction, indicated by high pressure, might contribute to bile duct proliferation, central venous distension, necrosis, and fibrosis.

Interestingly, a negative correlation between cystic volume and liver fibrosis grading was observed, though statistically insignificant. Pressure demonstrates a protective impact on the cyst wall but a detrimental effect on liver histology, given that ICCP directly correlates with liver parenchymal changes and inversely with cyst volume. Despite being smaller, high-pressure cysts exhibit more severe backpressure changes in the liver parenchyma. While this study's findings didn't attain significance, they hint at a potential trend that merits exploration in larger studies.

This study has implications for the management of choledochal cyst patients. Given our finding that smaller cysts are associated with high-grade liver fibrosis, prompt immediate surgery is necessary for smaller cysts. The findings underscore the urgency of timely intervention for smaller cysts to address associated complications effectively.

The strengths of the study lie in the meticulous measurement of cystic volume and ICCP, coupled with the comprehensive assessment of liver histology using the METAVIR scoring system. However, the study's limitations include its cross-sectional nature and the relatively small sample size, making the analysis and interpretation of the results difficult. Furthermore, the study did not correlate the findings with patient outcomes. Further research is needed to validate and expand upon these findings.

## Conclusions

This study unveils associations between cystic volume, ICCP, and liver fibrosis grading in choledochal cyst patients. Elevated ICCP is associated with increased hepatic fibrosis, as ICCP exhibits a direct correlation with alterations in liver parenchyma. Small-volume cysts, despite having high pressure, exhibit more severe backpressure, contributing to this fibrosis. These findings may guide future research and clinical management strategies, shedding light on potential avenues for therapeutic interventions in this complex congenital condition.
